# Detection of prions in the faeces of sheep naturally infected with classical scrapie

**DOI:** 10.1186/1297-9716-42-65

**Published:** 2011-05-18

**Authors:** Linda A Terry, Laurence Howells, Keith Bishop, Claire A Baker, Sally Everest, Leigh Thorne, Ben C Maddison, Kevin C Gough

**Affiliations:** 1 Animal Health and Veterinary Laboratories Agency, Woodham Lane, New Haw, Addlestone, Surrey, KT15 3NB, UK; 2 ADAS UK, School of Veterinary Medicine and Science, The University of Nottingham, Sutton Bonington Campus, College Road, Sutton Bonington, Leicestershire, LE12 5RD, UK; 3 ADAS UK, Department of Biology, University of Leicester, University Road, Leicester, LE1 7RH, UK; 4 School of Veterinary Medicine and Science, The University of Nottingham, Sutton Bonington Campus, College Road, Sutton Bonington, Leicestershire, LE12 5RD, UK

## Abstract

Classical scrapie is a naturally transmitted prion disease of sheep and goats. Contaminated environments may contribute to the spread of disease and evidence from animal models has implicated urine, blood, saliva, placenta and faeces as possible sources of the infection. Here we sought to determine whether sheep naturally infected with classical scrapie shed prions in their faeces. We used serial protein misfolding cyclic amplification (sPMCA) along with two extraction methods to examine faeces from sheep during both the clinical and preclinical phases of the disease and showed amplification of PrP^Sc ^in 7 of 15 and 14 of 14 sheep respectively. However PrP^Sc ^was not amplified from the faeces of 25 sheep not exposed to scrapie. These data represent the first demonstration of prion shedding in faeces from a naturally infected host and thus a likely source of prion contamination in the environment.

## Introduction

Prion diseases, or transmissible spongiform encephalopathies (TSEs), are fatal, neurological disorders that affect a range of mammalian species. They include scrapie in sheep, chronic wasting disease (CWD) in deer, bovine spongiform encephalopathy (BSE) in cattle and Creutzfeldt-Jakob disease (CJD) in humans. The "protein-only" hypothesis is the widely accepted paradigm for disease propagation and is based on the conversion of an apparently benign host prion protein (PrP^C^) into a pathological isoform, PrP^Sc^, which constitutes the infectious agent [1]. To date PrP^Sc ^is the only validated biochemical marker for prion diseases. These disorders are characterised by prolonged incubation periods before the onset of clinical signs, and the accumulation of PrP^Sc ^particularly within the CNS. The sites of PrP^Sc ^accumulation vary depending on the combination of host and prion strain under investigation, for instance classical ovine scrapie, cervid CWD and human vCJD display widespread distribution of PrP^Sc ^within the CNS and lymphoreticular tissues (LRS) [2-4] whereas for cattle BSE, atypical scrapie and human CJD the accumulation of PrP^Sc ^within LRS is much more restricted [5,6].

To date, studies investigating the shedding of the prion agent have focussed on scrapie and CWD. There is accumulating evidence for the excretion of prions within milk [7,8], urine [9,10], saliva [10-12] and faeces [13]. Such reports utilise methodologies that are exquisitely sensitive in their detection of prion infectivity or PrP^Sc^, namely bioassay or amplification of PrP^Sc ^by serial protein misfolding cyclic amplification (sPMCA). PMCA was pioneered by Soto et al. [14] in a rodent model of scrapie and this is an in vitro technique that amplifies trace amounts of PrP^Sc ^within a test sample during iterative rounds of sonication and incubation at 37°C. Importantly, this technique has been adapted for the high level amplification of PrP^Sc ^from natural sources of prion infection including ovine scrapie [15], bovine BSE [16], human CJD [17] and cervid CWD [18].

There is evidence from experimentally infected animals that prions are excreted in faeces. Safar et al. [13] used a rodent scrapie model to demonstrate the presence of prions in faeces when measured by conformation-dependent immunoassay (CDI) and transgenic mouse bioassay. Prions were present in faeces from hamsters throughout disease incubation. An analogous study in hamsters employed the sPMCA amplification of prions in faecal extracts and demonstrated the presence of prions during clinical disease [19]. In addition, experimentally infected mule deer excreted prion infectivity in faeces during preclinical and clinical stages of CWD [20].

Here, we apply sPMCA to investigate the excretion of prions within the faeces of naturally infected sheep, both in the preclinical and clinical stages of disease progression.

## Materials and methods

### Samples

Faecal samples were removed directly from the gut at post mortem or from the rectum of live animals while avoiding contamination with blood. Visible contamination with blood was not observed in any of the samples. This method of collection also minimised any contamination from the environment. Samples were stored at -20°C until assayed. Faeces were collected from sheep homozygous for VRQ (codons 136, 154 and 171 respectively of the *PRNP *gene) at 9 to 10 months of age and at clinical end-point (mean survival time of 22 months of age). As negative controls, faeces were collected from VRQ/VRQ unexposed sheep from a VLA New Zealand derived flock. No consistent differences in the consistency of the faeces were observed between exposed and negative control sheep.

### Faeces suspension preparation

Method 1 [Silicon dioxide (SiO_2_)]: After thawing, faecal pellets were transferred to 50 mL disposable tubes for homogenisation and diluted to three times the original mass using phosphate buffered saline (PBS, 15 mM KH_2_PO_4_, 81 mM Na_2_HPO_4_, 137 mM NaCl, and 3 mM KCl) containing 2× miniprotease inhibitors (without EDTA, Roche, Welwyn Garden City, UK). Each faecal suspension was vortexed for 5 to 10 min until homogeneous and 0.5 mL aliquots of this 33% faecal homogenate were transferred to a 15 mL falcon tube and stored at -80°C until required.

Method 2 [Sodium Phosphotungstic acid (NaPTa)]: After thawing the faecal samples were transferred to disposable tubes for homogenisation and diluted 1:9 in water containing protease inhibitors (Roche). The samples were then homogenised using the Prionics PrioGENISER™ (Prionics, Zurich, Switzerland) twice for 40 s at maximum speed. Prions were then extracted from faecal homogenates using NaPTa.

### Extraction of prions

Method 1 [SiO_2_]: Faecal homogenates (0.5 mL of a 33% w/v faecal homogenate) were diluted 1:1 in 2× phosphate buffer (pH 7) containing 1% deoxycholate (DOC) and 1% NP40, then further diluted to 5 mL with 1× PBS containing 0.5% DOC and 0.5% NP40. The faecal homogenates were then incubated for 1 h at room temperature (RT). Following centrifugation at 1500 *g *for 3 min the supernatant was removed and the pellet discarded. 80 μL of a 20% (w/v) SiO_2 _suspension were added to the 5 mL faecal supernatant and the sample rotated gently for 2 h at RT. The samples were centrifuged at 700 *g *for 4 min and the supernatant discarded. The SiO_2 _pellets were washed by gentle pipetting using 1 mL 0.1% SDS.

Method 2 [NaPTa]: Faecal homogenates (0.5 mL of a 10% w/v homogenate in deionised water containing protease inhibitors (mini-pill complete, Roche)) were transferred to homogenisation tubes and sodium dodecyl sulphate (SDS) added to a final concentration of 1%. The samples were then ribolysed (BioRad, Hemel Hempstead, UK) three times for 45 s each and cooled between each cycle. The samples were incubated at RT for 1 h rotating gently. Homogenates were then centrifuged for 20 min at 4°C at 15 000 *g *and the supernatants transferred to a clean tube. The pellets were discarded. The supernatant was diluted 1:1 with PBS containing 4% N-Lauroylsarcosine sodium salt (Sigma, Poole, UK) and treated with Benzonaze (1 unit/μL, Sigma) for 30 min at 50°C. Following treatment, NaPTa was added to a final concentration of 0.57% and the sample incubated at 37°C rotating gently for 30 min. Samples were centrifuged at 15 000 *g *for 15 min at 10°C and the supernatant discarded. Pellets were frozen at -80°C. Upon analyses, pellets were resuspended in 50 μL of PMCA buffer (PBS, additional 150 mM NaCl, 4 mM EDTA, pH8.0, 1.0% (v/v) Triton X-100 and miniprotease inhibitor, Roche).

### Amplification of prions

Method 1 [SiO_2_]: Faecal extracts derived from SiO_2 _precipitation were centrifuged at 16 000 g for 3 min. 10 μL of each faecal extract supernatant (from the equivalent of 8.3 mg wet weight of faeces) were mixed 1:9 with normal ovine brain homogenate. The samples were then subjected to 40 s cycles of sonication every 30 min incubating at 37°C for 24 h (1 round), after which the amplified samples were diluted 1:2 with PMCA substrate in a final volume of 100 μL and the sample subjected to further rounds of PMCA. Ten rounds of amplification were carried out. The amplification product was stored at -20°C. Samples from sheep incubating scrapie as well as from sheep not exposed to the scrapie agent were analyzed concurrently within the same run on the same sonicator. Each SiO_2 _extract was amplified within two separate experiments. After 10 rounds of amplification in the absence of PolyA no de novo synthesis was observed. Amplification was not extended beyond 10 rounds.

Method 2 [NaPTA]: Faecal extracts derived from NaPTa precipitation (8 μL; from the equivalent of 8 mg wet weight of faeces) were mixed 1:9 with normal ovine brain homogenate (obtained at post mortem from a VRQ/VRQ sheep from the New Zealand-derived VLA scrapie-free flock; a 10% (w/v) homogenate in PMCA buffer) supplemented with synthetic polyA RNA to a final concentration 100 μg/mL. The samples were then subjected to 40 s cycles of sonication every 30 min incubating at 37°C for 24 h (1 round). The sonicated product was then replenished 1:4 with fresh normal brain homogenate and sonication repeated for a further 24 h. This was repeated until four rounds were completed. Using this PMCA protocol rounds were restricted to 4 because de novo synthesis was observed at 6 rounds or more under PrP^Sc ^free conditions (data not shown). No de novo synthesis was observed in the presence of PolyA at round 4. The amplification products were stored after all PMCA rounds at -20°C until tested for PrP^Sc ^content.

### Analysis of PrP^Sc^

PMCA products were tested using the IDEXX HerdChek antigen test for scrapie as directed by the manufacturers. Positives were scored using the manufacturer's cut-off values. Alternatively, PMCA samples were digested with 50 μg/mL proteinase K, 0.045% (w/v) SDS for 1 h at 37°C before analysis by Western blotting using 12% (w/v) NuPAGE pre-cast Bis-Tris gels as previously described [21].

### Statistical analysis

When comparing the percentage of positive sPMCA reactions for different cohorts of samples, data were set up as 2 × 2 contingency tables and Fisher's exact test (one-tailed) was applied to derive *p*-values.

## Results

**Figure 1:**
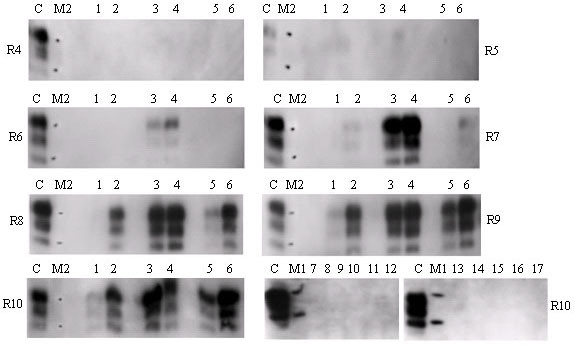
**Prion excretion in faeces of healthy and clinical scrapie infected sheep**. Prions extracted from 8.3 mg of faeces were used as seed for Method 1 [10 rounds of sPMCA]. After 4 to 10 rounds of sPMCA (as indicated), products were digested with proteinase K and 10 μL applied to Western blots. PrP was detected with monoclonal antibodies SHA31 and P4; molecular weight markers are shown (M1, 20 and 30 kDa; M2, 19 and 28 kDa). Sheep were either in the preclinical (lanes 1 and 2 for animals 1236/09 and 1305/09 respectively) or clinical stages of disease progression (lanes 3-6 for animals 0320/09, 0324/09, 0470/09 and 0572/09 respectively) or were unexposed to the scrapie agent (lanes 7-16 for animals R185, R223, R233, R234, R297, R303, R328, R382, R413 and R421 respectively). Two analyses were carried out for each sample and representative analyses are shown. Cerebellum (2.5 μL of a 10% w/v homogenate) from a terminally affected sheep was analysed on each blot as a PrP^Sc ^control (C).

Faeces were sampled directly from the transverse colon of clinically-affected sheep at post mortem or from the rectum of live sheep with preclinical scrapie. Prions were extracted from faeces employing a mild detergent extraction (0.5% w/v DOC and NP40) followed by SiO_2 _precipitation. Prions extracted using SiO_2 _were amplified over 10 rounds of sPMCA before attempted detection of PrP^Sc ^by Western blot. This method was previously shown to amplify prions from the saliva of sheep incubating scrapie [11]. Here, when applied to the faeces from scrapie infected animals, PrP^Sc ^was amplified from 6 of 29 animals: four animals with clinical disease and two animals which were clinically normal at approximately the mid-point of disease incubation (10 positive sPMCA reactions from 58 analyses; Figure 1, Table 1). No prions were amplified from faeces collected from sheep which had not been exposed to the scrapie agent (10 animals, 60 analyses, Figure 1, Table 1). These data demonstrate that prions were secreted in faeces from sheep incubating natural scrapie (*p *= 0.001).

**Table 1:** Amplification of PrP^Sc ^from sheep faeces

Origin of faecal sample	Animal ID	**number positive/total number of analyses Method 1**^**a**^	**number of positive/total number of analyses Method 2**^**b**^
	R382	0/6	0/2
	
	R328	0/6	0/2
	
	R234	0/6	0/2
	
	R413	0/6	0/2
	
	R421	0/6	0/2
	
Unexposed sheep	R185	0/6	0/1
	
	R223	0/6	0/1
	
	R233	0/6	0/1
	
	R297	0/6	0/1
	
	R303	0/6	0/1
	
	**Total**	**0/60**	**0/15**^**c**^

	1203/09	0/2	2/2 (4,4)
	
	1236/09	1/2 (9)^d^	1/2 (4)
	
	1252/09	0/2	2/2 (4,4)
	
	1271/09	0/2	2/2 (4,4)
	
	1284/09	0/2	2/2 (4,4)
	
	1292/09	0/2	2/2 (4,4)
	
Preclinical exposed sheep age 9-10 months	1298/09	0/2	1/2 (4)
	
	1305/09	2/2 (7,10)	1/2 (4)
	
	1227/09	0/2	1/1 (4)
	
	1279/09	0/2	1/1 (4)
	
	1286/09	0/2	1/1 (4)
	
	1296/09	0/2	1/1 (4)
	
	1299/09	0/2	1/1 (4)
	
	1307/09	0/2	1/1 (4)
	
	**Total**	**3/28**	**19/22**

	317/09	0/2	0/2
	
	319/09	0/2	1/2 (4)
	
	320/09	2/2 (6,6)	1/2 (4)
	
	324/09	1/2 (6)	2/2 (3,4)
	
	421/09	0/2	0/2
	
	352/09	0/2	0/2
	
	353/09	0/2	0/2
	
Sheep with clinical scrapie	354/09	0/2	0/2
	
	359/09	0/2	1/2 (4)
	
	382/09	0/2	0/2
	
	457/09	0/2	0/2
	
	470/09	2/2 (8,9)	2/2 (4,4)
	
	606/09	0/2	0/1
	
	608/09	0/2	1/1 (4)
	
	572/09	2/2 (7,9)	0/1
	
	**Total**	**7/30**	**8/27**

^a^Method 1: Prion was extracted in NP40 and DOC, precipitated with SiO_2 _and amplified by sPMCA for 10 rounds.

^b^Method 2: Prions were extracted in SDS, NaPTa precipitated and amplified by sPMCA in the presence of polyA for 4 rounds.

^c^Faeces from a further 15 animals was analysed in duplicate by this method and no prions were detected (data not shown).

^d^The sPMCA round at which the sample first became positive is indicated in parenthesis.

In order to estimate the levels of scrapie prions present within faeces, the limit of detection of the methodology was determined for the earliest sPMCA round that amplified PrP^Sc ^from both preclinical and clinical faecal samples. Using SiO_2 _extraction coupled with sPMCA amplification of prions in the absence of polyA RNA, seven rounds of sPMCA amplification yielded detectable PrP^Sc ^for 4 of the samples, 3 taken from clinical animals (0320/09, 0324/09, 0572/09) and one taken from an animal in the preclinical stage of disease (1305/09). A dilution series of a brain homogenate from an animal with terminal scrapie was spiked into a faecal homogenate and then extracted and amplified. Extraction followed by amplification for 7 rounds of sPMCA detected PrP^Sc ^down to 0.64 ng of brain material spiked into 167 mg wet weight of faeces (data not shown).

To investigate further the shedding of prions in faeces, a second method of extraction and amplification was developed. This method used potentially more stringent extraction conditions and enhanced sPMCA amplification. Briefly, prions were eluted from faeces with 1% SDS followed by NaPTa precipitation of prions and sPMCA amplification employing polyA;-the presence of polyA having been shown to increase the sensitivity of the sPMCA amplification [15,22]. The presence of PrP^Sc ^was demonstrated in the amplification product using the IDEXX HerdChek antigen assay for scrapie (Table 1). PrP^Sc ^was detected in the faeces of all 14 sheep which were in the preclinical stages of scrapie and sampled at 9 to 10 months of age (19 positive sPMCA reactions from 22 analyses; Table 1). Of the sheep at clinical end-stage, PrP^Sc ^was amplified from the faeces of 6 out of 15 sheep (8 out of 27 analyses were positive). Analyses of animals not exposed to the scrapie agent again demonstrated that no prions could be detected (25 animals, 45 analyses; Table 1 and data not shown). These data indicate that scrapie prions are excreted in faeces in both preclinical (*p *< 0.001) and clinical (*p *< 0.001) stages of the disease. PrP^Sc ^was detected at the earliest following round 4 using this method except in 1 sample (324/09) where PrP^Sc ^was detected following round 3.

These data report for the first time the excretion of prions in the faeces of naturally infected animals and further support the role of the faecal-oral route as a means of horizontal transmission in the field.

## Discussion

The reported study demonstrates the presence of prions in the faeces of sheep naturally infected with scrapie. Additionally, PrP^Sc ^was present in the faeces of sheep both in the terminal stages of disease and when in the early preclinical phase, suggesting that prions are likely to be shed into the environment throughout pathogenesis. These data concur with a study of mule deer experimentally infected with CWD [20] where infectivity was observed in faecal samples collected from the deer from nine months after inoculation and maintained during the clinical phase several months later. Together these data are fully consistent with faeces being a source of environmental prion contamination that contributes to transmission for both scrapie and CWD.

Within the present study, two distinct methods were applied to detect faecal PrP^Sc^. Prions were detected less frequently when employing mild detergent extraction, SiO_2 _precipitation and a standard sPMCA amplification, compared to when using SDS extraction, NaPTa precipitation and sPMCA incorporating polyA. This may indicate that the latter method has an improved sensitivity. The method based on mild extraction and non-enhanced sPMCA detected prions more frequently in clinical samples and in contrast the method based on SDS extraction and polyA-enhanced amplification detected prions more frequently in preclinical samples. This suggests that preclinical animals may be more likely to secrete prions compared to animals with clinical disease, but that the levels of prion present within faeces is higher in the clinical animals. With the mild extraction conditions amplification of prions was observed at earlier rounds in samples from clinical compared to preclinical animals, supporting the proposition that prions are at higher levels in faeces during clinical disease.

Several sources of prions in faeces could be postulated including environmental ingestion and swallowing of infected saliva [11]. However, the most likely source is shedding from the gut-associated lymphoid tissue. Early and rapid accumulation of PrP^Sc ^in the Peyer's patches is consistent with the detection of prions in faeces during the preclinical phase. In sheep with a VRQ/VRQ *PRNP *genotype this accumulation commences at around 2-3 months of age. Ruminants have specialised Peyer's patches that are continuous throughout the length of the ileum amounting to approximately 100 000 follicles and all of these could potentially be infected and shedding prions into the lumen. However the number of follicles diminishes at around 12-15 months of age thus reducing the potential for shedding at this site in older sheep such as those with clinical disease in the present study. Additionally, the recto-anal mucosa associated lymphoid tissue may also play a role in contributing to the levels of PrP^Sc ^found in the faeces; in the present study this route would not contribute to prion in the samples from clinical sheep due to direct sampling from the gut. These factors would be expected to result in lower prion levels and/or a lower frequency of prion excretion within the clinical faecal samples. However, this is not supported by the presented data indicating that levels of prion excretion is not solely influenced by excretion from RAMALT or the ileal Peyer's patches.

Not all prion diseases result in infection of the gut-associated lymphoid tissue. The low levels of prion infection in the gut of cattle with natural BSE [23] could explain the apparent, fortuitous failure of lateral spread of BSE within herds.

Here, with the estimated levels of prion shed within the faeces combined with an estimated ovine faecal output of 67 kg/month [24] it could be speculated that between 9 months and 22 months of age sheep could excrete prions equivalent to the potentially infectious load within approximately 3.3 mg of clinically infected brain. Tamguney et al. [20] used bioassay to estimate the levels of infectivity in faeces from experimentally-infected CWD deer; over a 10 month period these levels were estimated to approach that present within a clinically infected whole brain. These levels are much higher than those found in the present study with scrapie infected sheep; however, this could reflect differences between the prion strains and host species under investigation or be due to differences in the prion detection methodologies employed.

Previous studies have indicated that risk factors for horizontal transmission in the field involve contact during the postnatal period [25-27]. However, exposure to pasture that has been grazed by infected sheep is sufficient to transmit disease even in the absence of lambing [25,28]. The findings of the present study would suggest that prions in faeces will contribute to this contamination and support the role of the faecal-oral route as a means of horizontal transmission. Strategies for the control of scrapie will need to take this into account particularly for goats where successful elimination is still reliant on depopulation, disinfection and restocking.

In North America, the presence of prions in secreta and excreta of deer and the resultant contamination of the environment is likely to have contributed to the spread of CWD and this is likely to progress unabated given the estimation of the amount of prions contained within cervine faeces [20]. Therefore environmental contamination of pasture remains a serious impediment to future eradication of prion disease in both Europe and in North America.

## Competing interests

The authors declare that they have no competing interests.

## Authors' contributions

LT contribute to the design and coordination of the study and prepared the manuscript. LH carried out all experimental work for method 2. KB carried out all experimental work for method 1. CB carried out method development work for method 1. SE contributed to the coordination of the study and analyses of results from method 2. LTh initiated and designed the PMCA studies. BM contributed to the coordination of the study and analyses of results from method 1. KG contributed to the coordination of the study and analyses of results from method 1 and assisted in manuscript preparation. All authors read and approved the final manuscript.
